# Continuous Monitoring of Pigs in Fattening Using a Multi-Sensor System: Behavior Patterns

**DOI:** 10.3390/ani10010052

**Published:** 2019-12-26

**Authors:** Miguel Garrido-Izard, Eva-Cristina Correa, José-María Requejo, Belén Diezma

**Affiliations:** 1 Laboratorio de Propiedades Físicas y Técnicas Avanzadas en Agroalimentación. ETSIAAB, Universidad Politécnica de Madrid, Avda. Puerta de Hierro 2, 28040 Madrid, Spain; evacristina.correa@upm.es (E.-C.C.); josemaria.requejopuerto@hendrix-genetics.com (J.-M.R.); belen.diezma@upm.es (B.D.); 2 Hypor, a Hendrix-Genetics Company. Hypor B.V.Villa ‘de Körver’, Spoorstraat 69, 5831 CK Boxmeer, The Netherlands

**Keywords:** individual temperature logger, electronic feeding station, phenotyping, feeding patterns, circadian rhythm

## Abstract

**Simple Summary:**

The livestock sector seeks technologies and procedures to collect and manage data and information about its facilities and animals being the basis of the so-called precision livestock. The installation of unusual devices in commercial facilities, as well as the use of electronic feeding stations, allows observers to characterize the behavior pattern of each individual in order to improve farm management techniques and, therefore, its productivity. In this study, 30 Landrace pigs were monitored during the whole fattening period. Results from the study show that the ear skin temperatures of the animals can be used to distinguish animals with different thermal patterns. The parameters extracted from the feeding stations show consistent relationships between the parameters related to the frequency, size, and duration parameters, highlighting the differences in the feeding strategies.

**Abstract:**

In this work, a complete fattening period (81 days) of a total of 30 Landrace pigs housed in two pens of a nucleus in Villatobas (Castilla-La Mancha, Spain) were supervised. The ear skin temperature of each animal was recorded every three minutes. The body weight, the date, the duration, and the amount of feed consumed per animal was monitored via an electronic feeding station. The objective was the identification of animals with different behaviors based on the integration of their thermal and intake patterns. The ear skin temperatures of the animals showed a negative relationship between the mean and the standard deviation (r = 0.83), distinguishing animals with different thermal patterns: individuals with high-temperature values show less thermal variability and vice versa. Feeding parameters showed differences in the feeding strategies of animals, identifying fast-eating animals with a high rate feed intake (60 g/min) and slow eaters (30 g/min). The correlation between the change in the rate of feed intake along with animal growth and feed efficiency reached a significant negative value (−0.57), indicating that animals that do not alter their rate of feed intake along breeding showed higher efficiencies. The difference in temperature of an animal with respect to the averaged group value has allowed us to identify animals with differentiated feeding patterns.

## 1. Introduction

At present, the management of livestock farms has to integrate profitability criteria and aspects related to animal welfare and health, being obliged to reach increasingly complex compromise solutions. In this situation, the sector seeks technologies and procedures to collect and manage data and information about its facilities and animals that may be the basis of the so-called precision livestock. Thus, the concept of massive phenotyping of animals arises, in which the registry of significant parameters (temperature, movements, sounds, etc.) related to aspects of well-being, health or productivity is sought.

Special attention has been given to the measurement of surface temperature in different species of mammals as an indicator of the level of stress or the existence of diseases [1]. More recently, it has been shown that surface temperature monitoring in pigs can give indications of their thermo-regulatory effort [2] under normal conditions, without stress. On the other hand, other results support that [3] heat production in pigs during fattening is associated with physical activity, thermal effect of intake and basal metabolism, which together with the fact that animals with higher body temperatures invest more energy in the production of metabolic heat at the expense of productivity, allows the sector to establish that animals with low physical activity and heat production show better productivity [4]. However, there are still uncertainties in the assumption that the measured surface temperature can be taken unfailingly to estimate different animal-based parameters. Regarding infrared thermal imaging, which is one of the most widespread techniques for determining surface temperature [5], limitations on accuracy have been noted, due, for example, to the movement of animals [6], to the values of the emissivity of the skin surface applied [7], or to the effect of environmental temperature [8].

Behavior patterns are defined as possible modes of action in a given situation or condition, including activity level, intake patterns, and interactions between animals. In numerous research papers, behavior patterns are studied through image registration, with complex and expensive analysis procedures [9,10] or through the incorporation of accelerometers [11] or sound recorders [12]. In any case, all these systems involve the installation of unusual devices in commercial facilities. The electronic feeding stations, systems installed in numerous farms, allow them to characterize the patterns of intake of each individual by means of the recognition of radiofrequency identifiers (Radio Frequency Identification, RFID) and including in the variable register the time of the event, the frequency of visits, the duration of the visit, the intake per visit, and the weight of the animal. In commercial farms, this information is used to determine the overall efficiency of each animal, especially in certain areas dedicated to genetic improvement [13,14]. 

Previous results have sought to identify different feeding strategies for growing pigs through the analysis of feeding station records [15]. The feeding parameters are the complex result of many factors that act simultaneously. Thus, breeding [16,17], hour of the day [18], age and social and environmental conditions influence their values [15,19]. Despite this, the feeding parameters, such as the total feeding time, the number of visits to the station or the rate of feed intake allow speculation of a variety of eating patterns from *nibblers* to *meal eaters*, the number and time of visits [20], and *fast* to *slow* eaters according to the rates of feed intakes. Moreover, [15], an hypothesis that the animals with the highest daily gain are those that show “*meal eater* and *fast eater*” strategies, which had already been reported by [21], who indicated that pigs that eat faster also eat more and grow faster, although they have no greater or lower residual feed intake. Similarly, [16] showed that neither feed intake, nor time in the feeding station, nor feed intake related to feed conversion. 

This paper proposes the implementation of surface temperature recorders and electronic feeding stations as monitoring tools for each animal during a period of complete fattening. The objective is the identification of animals with different behaviors based on the integration of their thermal and intake patterns. 

## 2. Materials and Methods 

### 2.1. Animals

Between 17 April and 6 July 2018, 30 Landrace pigs (all males) were monitored, covering their entire fattening period. The animals were distributed equally in two boxes (15 m^2^/box) with full concrete slats in a breeding farm in Villatobas, Castilla-La Mancha, Spain (39°54′02.7″ N 3°17′31.4′′ W) belonging to the company Hypor, an important provider of swine genetics and part of the Hendrix Genetics corporation, Boxmeer, The Netherlands. 

The environmental temperature was automatically controlled by an air cooling system (RN 12, Exafan, Spain). The extractors, two fan chimneys located at the opposite end of the room entrance, removed excess hot air from the boxes when the temperature exceeded the setpoint (20 °C + 4.0 °C). The hot air from outside was introduced through the cooling units (wet cellulose surface, located at the corridor) to reduce the temperature and increase the humidity in the pens. The cool and wet air enters the pens through an opening on the top of the walls. To verify the cooling system, a specific temperature sensor is located 2 m high at the center point of the room (with six pens). Each room has two windows connected to the corridor. The lighting schedule, according to the state of the lights in the room, was 14 h of light and 10 h of darkness, considering the daytime from 7:00 a.m. to 9:00 p.m. and night from 9:00 p.m. to 7:00 a.m.

**Figure 1:**
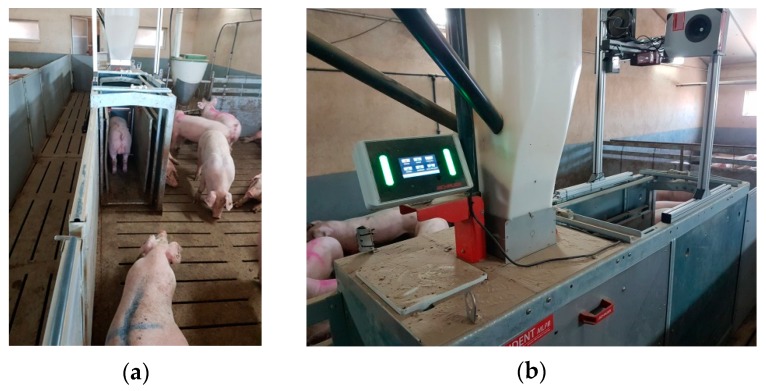
(**a**) Weighing scale and (**b**) feeding station used during the test.

All animals had access to two nipples to drink and *ad-libitum* dry food through an electronic feeding station (Compident MLP, Schauer Agrotronic GmbH, Austria), which weighed the food in the feeder before and after the visit, providing the intake value (Figure 1). Access to the feeding system is controlled by the unique identification of each animal through the RFID marker located on the tag. In this way, the animal’s code, date, time, and amount of food consumed are recorded and stored in a single database at each visit. It should be noted that at the beginning of the test, the intake of the animals is not monitored, eating is *ad libitum*. Once the animals have adapted to the machines (learning period), the feeding stations began to function properly (monitoring intake). 

The information provided by the feeding station was added with the weight of each animal at each visit, which like the station data, were collected through the identification of the animal and using an automated weighing scale (Figure 1).

Table 1 summarizes the most relevant test information for the 23 animals finally considered, removing those that were discarded during the test. The causes of these discards were bitten by the tail of the animal by other individuals, loss of sensor and tag used to carry out the monitoring; or sensor damage.

### 2.2. Temperature Measurements 

**Figure 2:**
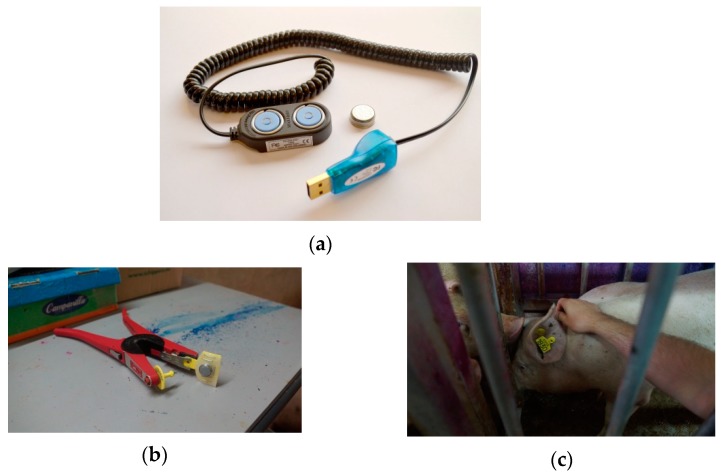
(**a**) DS1992 iButton sensor and DS1402D-DR8 reader; (**b**) detail of the coupling of the iButton sensor to the barcode tag; and (**c**) installation of the tag with the sensor attached to the animal to be monitored.

The iButton DS1922E loggers (Dallas Semiconductor, USA), data acquisition devices with an integrated temperature sensors with factory calibration were used to record the ear skin temperature (EST) of each animal during the fattening period (Figure 2a). Both the data transfer and the configuration of the thirty sensors used (Table 2) were performed using the reader “DS1402D-DR8” (Figure 2a) and the software “OneWireViewer version 3.17.44” provided by the distributor.

The boars are identified with a tattoo, a barcode tag, and RFID. Each iButton was fastened to an additional barcode tag on the ear of each animal in such a way that it was in contact with the inner part of the ear (Figure 2b,c).

Six iButton DS1923 loggers (Dallas Semiconductor, Dallas, TX, USA), similar to those used for animal monitoring, but with relative humidity sensor added, were used to record the temperature and humidity (with factory calibration) conditions of the environment where the animals were during the entire fattening period. In the same way as for the loggers attached to the tag, both the data transfer and the configuration (Table 2) were performed via a wire using the “DS1402D-DR8” reader and the “OneWireViewer version 3.17.44” software provided by the distributor.

**Figure 3:**
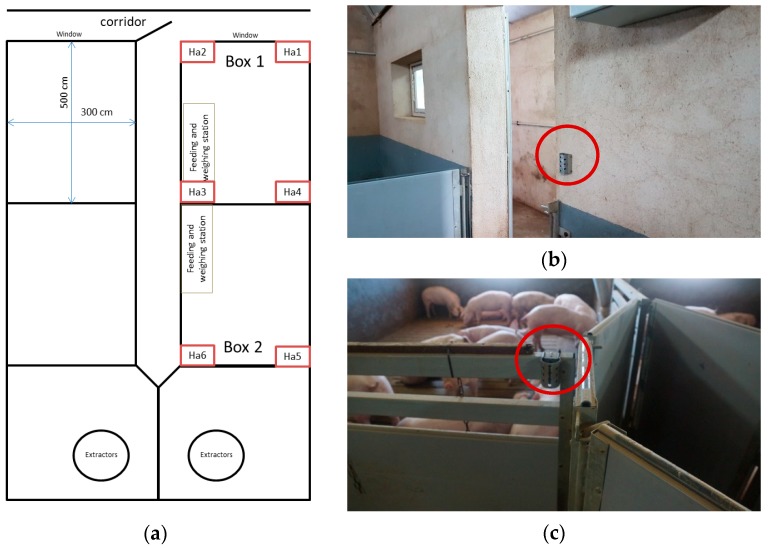
(**a**) Distribution plan of the six environmental sensors (from Ha1 to Ha6). (**b**,**c**) Detail of the installation of two loggers (Ha2 and Ha6 on the map), earmarked by a red circle are the environmental loggers.

Each of the six iButtons used was integrated into a protective structure made on a perforated (allowing free air circulation) stainless steel sheet of 3 mm thick, open at the top and bottom sides, distributing them evenly throughout the two boxes under study at a height of 1.15 m (Figure 3). 

### 2.3. Data Analysis 

The analysis of the data recorded by the loggers used as well as from the feeding stations were performed with MatLab R2018b software (MathWorks, Massachusetts, USA). In the following paragraphs, the statistical analyses performed according to their appearance in the document are explained.

Raw data obtained from both environmental (*n* = 10,179 data points from each logger) and EST (*n* = 19,145 from each logger), were interpolated at the time base of one of the EST loggers. The elaboration of this interpolation to a single time base of all the loggers (both environmental and EST) allowed us to adjust data size (*n* = 19,145).

Descriptive statistics of environmental parameters (temperature and relative humidity) and skin temperature of the pigs were calculated for the purpose of assessing the independence of the fluctuations of both series.

Correlations between the mean and the standard deviation of the time series of the temperature of each animal were calculated by the use of the *corrcoef* function. This function returns the matrix of correlation coefficients and the matrix of *p*-values for testing the hypothesis that there is no relationship between the observed phenomena (null hypothesis). If an off-diagonal element of *p* is smaller than the significance level (default is 0.05), then the corresponding correlation in *r* is significant.

Raw data obtained from electronic feeding stations (*n* = 30,718) were filtered by removing those records corresponding to discarded animals (7 pigs). The weights recorded from this resulting electronic feeding stations data (*n* = 24,912) were adjusted, in order to clear the erroneous records. Weights data equal to zero and anomalous excess (consecutive weights with a difference greater than 10 kg) were eliminated. Then, a first outlier detection was performed by the use of *isoutlier* function. An outlier was defined as a value that exceeds more than three times the local median, in a 10-point window. Each outlier detected were replaced by a new value, obtained from the interpolation of the a priori and a posteriori weights closest to the time of registration of the outlier value. Then, a second and finer outlier detection was performed (a value that exceeds more than three times the local median, in a 5-point window), replacing in the same way as before, the outliers detected. Adjustment line (one per animal) was performed on the resulting smoothed weight records.

Once animals’ weight was corrected, parameters for the electronic feeding station were evaluated taking only into account the visits in which the intake was higher than 15 g. Correlations among the variables computed from the feeding station were calculated (number of visits [NV], total visit time (s) [TVT], Average visit time (s) [AVT], Total intake (kg) [TI], Average intake per visit (kg) [AIV], total intake rate (g/s) [TIR], weight gained (kg) [WG] and efficiency (kg gained/kg intake) [E]). The evolution of animals over time is visualized by different scatter plots.

A new series of data defined as the difference between the instantaneous temperatures of each animal with respect to the averaged group temperature value at that time (ΔT) has been calculated. Three-day averages of both thermal parameters (including ΔT) and intake parameters have been considered, and it has been calculated the correlations between them for each animal.

## 3. Results and Discussion

### 3.1. Animal Temperature vs. Environment Temperature and Day-Night Cycles 

**Figure 4:**
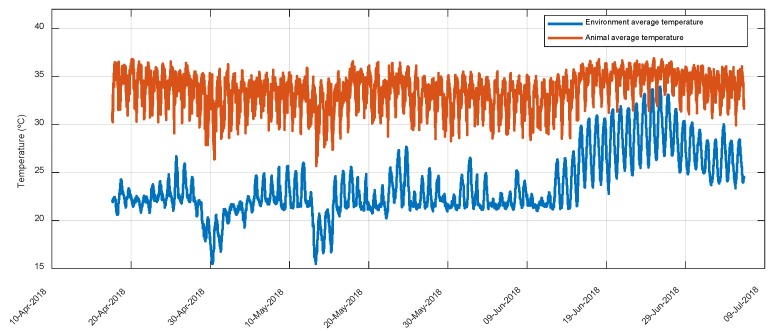
Time series of the average temperatures of the environment and of the animals recorded during the test.

**Figure 5:**
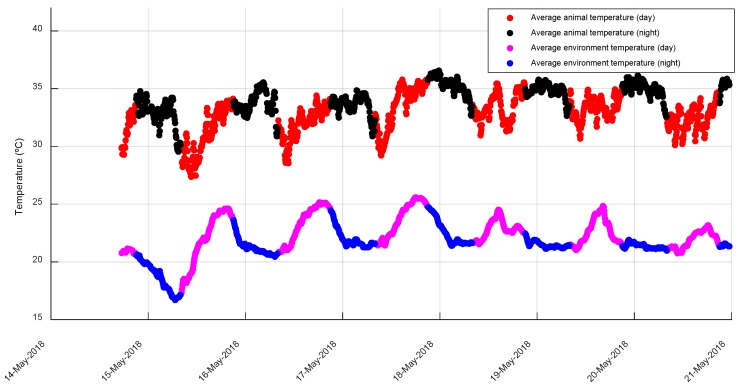
One week detail of the time series of the average temperatures of the environment and animals; the corresponding time and day sections are indicated by different colors.

**Figure 6:**
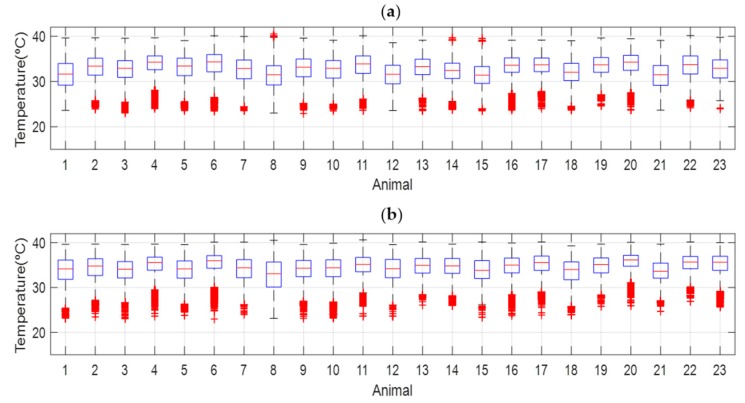
Boxplot of the (**a**) day and (**b**) night temperature for each monitored animal. Central mark indicates the median, and the bottom and the top of the edges of the boxes indicate the 25th and 75th percentiles respectively. The whiskers extend to the most extreme data points not considered outliers, and the outliers are plotted individually using the ’+’ symbol.

Figure 4 includes the historical series of the average temperatures of the environment and animals. EST is consistently maintained above the environment temperature. Figure 5 shows the detail of this historical series for a period of one week; the independence of the fluctuations of both series is observed, which indicates that the sensors of the ear tags are mostly affected by the body temperature of the animal. The average EST during the night was 34.84 ± 0.79 °C and during the day 32.80 ± 1.0 °C (Figure 6); while the behavior in the ambient temperature is the reverse: the average of the medians during the day was 23.22 ± 0.71 °C, with a relative humidity of 52.60 ± 2.48% and during the night 22.25 ± 0.82 °C, with a relative humidity of 55.14 ± 2.12%.

### 3.2. Animal Temperature

**Figure 7:**
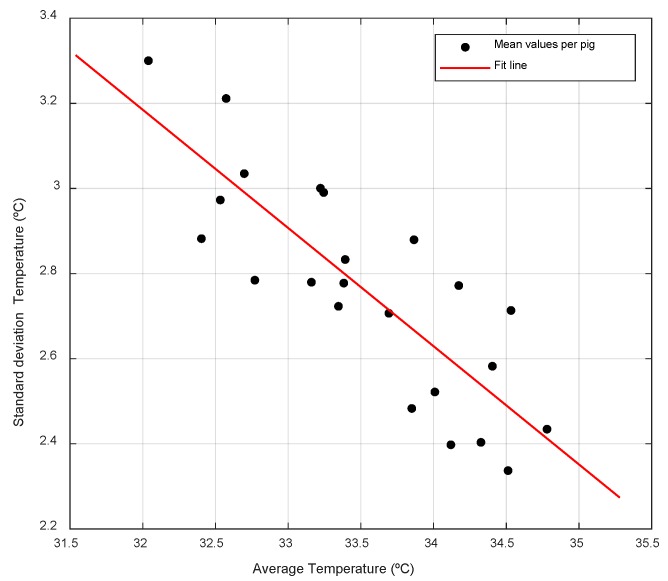
Ratio of the mean and standard deviation of the temperatures recorded during the test per animal (r^2^ = 0.69; y = −0.28x + 12).

**Figure 8:**
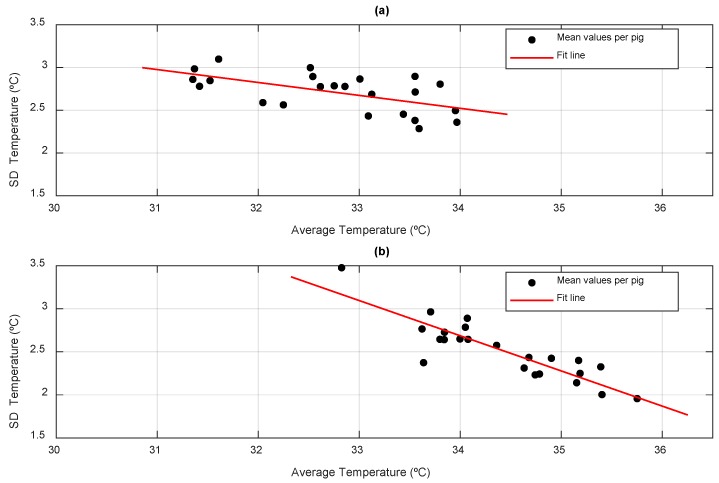
Ratio of the mean and standard deviation of the temperatures recorded separating (**a**) day (r^2^ = 0.35; y = −0.15x + 7.7) and (**b**) night (r^2^ = 0.77; y = −0.41x + 16.6).

Figure 7 represents the average temperature and the standard deviation of each of the animals monitored. There is a significant linear relationship with a coefficient of determination of 0.69 between the mean and the standard deviation of the time series of temperatures of each animal. So that the animals with higher average temperatures show lower variations of the recorded temperatures, which had been previously observed in previous works with shorter supervision periods. This corroborates that the time series of temperatures is a tool that allows differentiation between animals according to their thermal patterns [14]. Figure 8 shows the same representation distinguishing between day and night. It is observed that the relationship between average temperature and standard deviation is accentuated during the night (r^2^ = 0.77), at which time the animals have less general activity as well as intake (see Section 3.4 Behavior Patterns), so that the surface temperature of the animals may be more related to the basal metabolism [3]. 

### 3.3. Electronic Feeding Stations

**Figure 9:**
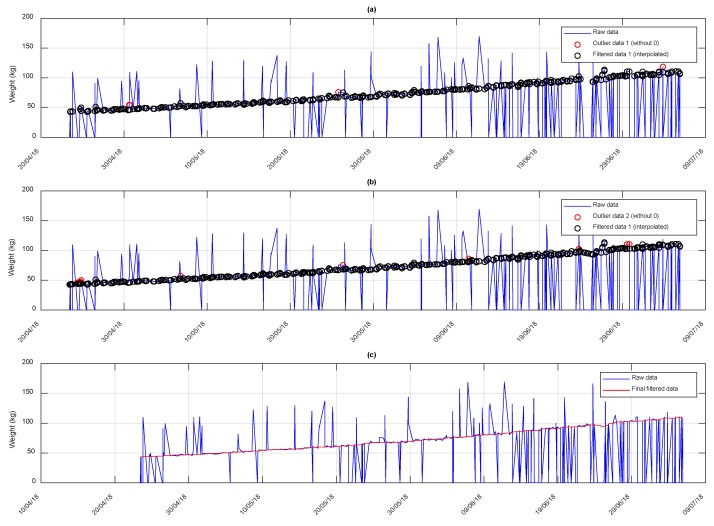
Filtration process of the weight scale records during the test for the animal with tag 6229: (**a**) original weight data and outliers detection by over a sliding window of length 10; (**b**) original weight data and outliers detection by over a sliding window of length 5; and (**c**) original weight data and adjustments results.

As an example, Figure 9 shows the record of the weight of an animal at each visit to the feeding station throughout the entire test. It is observed that there are erroneous records (by default and by excess) that have to be cleared before further analysis. Figure 9 shows the adjustment to the weight data as a function of the date of registration. Weights data equal to zero and anomalous excess were eliminated and were defined as outliers, in explanation, points that exceed more than three times the local median, in a 10-point window (Figure 9a), and then in a 5 point window (Figure 9b). Once these points have been eliminated, the adjustment is made and the value of the adjustment function is assigned to all registered points, both those that have been eliminated as outliers and those that have not (red line in Figure 9c). The average error of the adjustments made in the weight records of all animals is 2%.

Only the visits in which the intake was higher than 15 g were considered for further analysis, as other authors [17] have suggested it. Non-feeding visits were 9.54% of all visits, with an average time of 20.6 s (7.4% of the mean duration of feeding visits). The average values per animal of the variables obtained at the feeding station are included in Table 3. The variable number of visits, the average time of visits and the average intake per visit are the variables that show the greatest variability among animals (coefficients of variation of 57%, 40%, and 45%, respectively). While the variables of total intake and weight gain have lower coefficients of variation (11%). The coefficient of determination between the total intake and the weight gained is 0.8, which is in line with the small variability found in the efficiency values (a coefficient of variation of 5% and a range between 0.38 and 0.47). These efficiency values are within the usual ranges in the fattening phases, although there are works in which efficiencies as low as 0.082 have been found under conditions similar to the present study and for animals of about 65 kg [14].

### 3.4. Behavior Patterns

The correlations between feeding parameters shown in Table 4 reveal, as it was already demonstrated by [15], the high correlation between the parameters related with the frequency (number of visits), size (total intake, average intake per visit), and duration (total visit time, average visit time) parameters. The average time per visit had a high negative correlation with the number of visits (r = −0.81), as well as with the average intake per visit (r = 0.9). The number of visits had a high negative correlation with the average intake per visit (r = −0.84). In addition, total visit time had a high negative correlation with total intake rate (r = −0.83), while the correlation between weight gained and total intake was positive (r = 0.88). 

Although differences in feeding patterns have already been analyzed between different breeds of pigs [15], in our study, even though all the monitored pigs belong to the same breed (Landrace), it can be observed in Table 3 the large feeding pattern differences. Table 3 shows that visits were more frequent and shorter for the pig tagged as 6219 than for the pig tagged as 6228, having both very similar total intake and total visit time values (175.2 kg vs. 176.2 kg and 171,262 s vs. 175,488 s respectively); these pigs, using the nomenclature by Fernandez, could be identified as *nibbler* and *meal eater*. On the other hand, pigs 6221 and 6225 could be named *fast* and *slow* eaters, respectively, applicable to the total rate feed intake. 

Despite the disparity in the number of visits between animals, it is found that weight gain, total intake, and, therefore, efficiency is very similar between them at the end of the supervised period (Table 3), indicating that the *ad libitum* feeding system through automated stations dampens the effect that feeding patterns can have on the efficiency of these animals.

**Figure 10:**
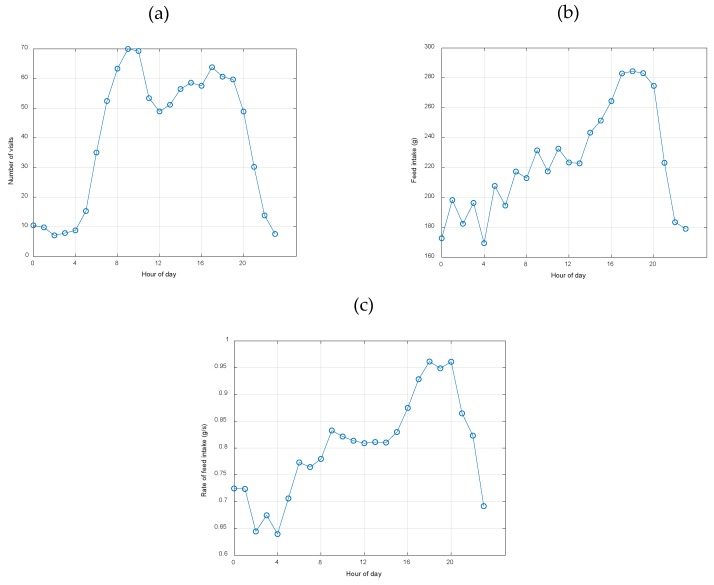
Diurnal distribution of (**a**) number of visits, (**b**) feed intake and (**c**) rate of feed intake parameters.

In relation to circadian rhythm, Figure 10 shows the distribution of visits, feed intake, and the rate of feed intake throughout the day. As seen, the circadian rhythm of the number of visits (Figure 10a) was characterized by two peaks: around 09:00 and 17:00. When the circadian rhythm of the number of visits was compared with the daily distribution of the feed intake and rate of feed intake (Figure 10b,c) the afternoon peak of the number of visits coincides with the fastest rate of feed intake and the highest feed intake. Thus, the visits throughout the morning would be considered of low productivity, in terms of intake. During the nighttime, visits and feed intake were infrequent, decreasing also the rate of feed intake.

The appearance of a faster rate of feed intake during the afternoon as well as the night behavior detected corroborates what has been said in previous studies [15].

**Figure 11:**
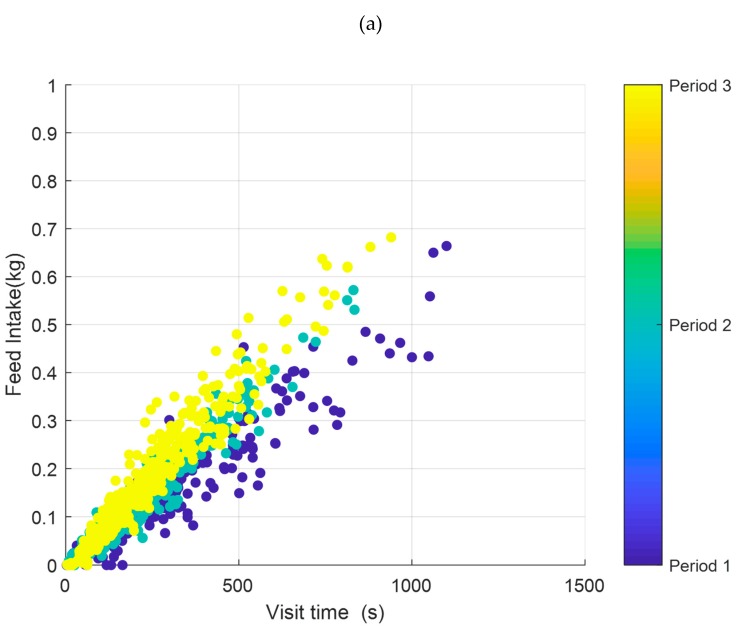

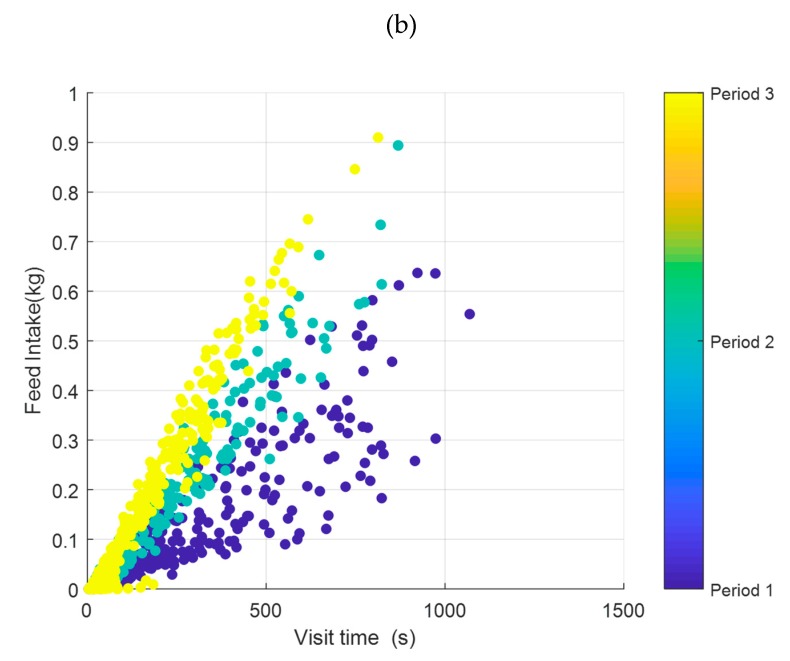
Feeding behavior in terms of visit duration and feed intake during the different periods for pigs tagged as (**a**) 6212 and (**b**) 6215.

Figure 11 shows the visit time vs. the feed intake in each visit during the entire fattening period for pigs tagged as 6212 and 6215; the different colors of points correspond to the three growth periods. The fattening period was divided into three periods of equal duration. The slope of the fit lines to each period represents the rate of feed intake. In general, the rate of feed intake increases with the age of the animals, on average from 38 g/min for the first period until 71 g/min for the last period. However, it appeared there were differences between the animals (Figure 11). Some of them presented a low evolution in their rate of feed intake (increase below 20 g/min, Figure 11a) and others a very high increase (more than 40 g/min, Figure 11b). This shift towards fast eater strategies could be associated with a progressive increase in the weight of each animal, which has been reported by previous works [22]. 

**Figure 12:**
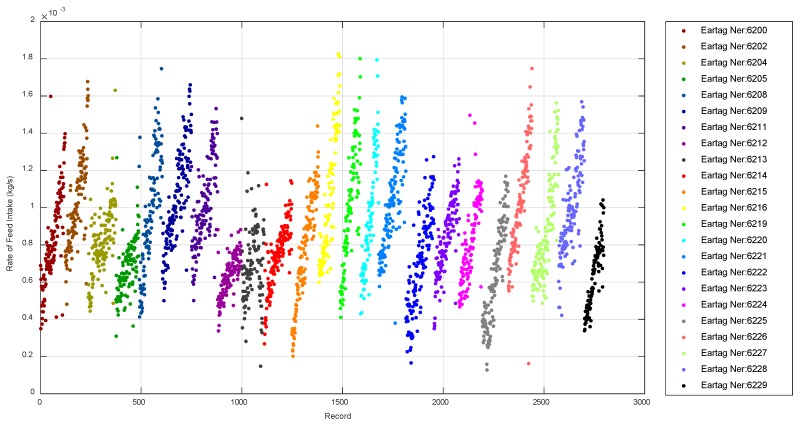
Evolution of the rate of feed intake during the whole fattening period for each monitored animal.

In an attempt to systematize the visualization of the observed changes in the rate of feed intake along the fattening period and thus, be able to identify different feeding patterns based on this variability, Figure 12 is proposed. Figure 12 shows the rate of feed intake at each day and night interval along the fattening period for each of the animals (denoted by different colors). A linear fit was applied to the points of each animal; the slope of the adjustment represents a kind of acceleration of the rate of feed intake (increment of the rate of feed intake/increment of time).

Table 5 shows the correlations between the slopes of the feed intake vs. time adjustment lines (Figure 11), acceleration of the rate of feed intake, weight gained, and feed efficiency. The slope in the second period shows a high positive correlation with the slopes in the other periods, while the other two are less correlated between them, which shows that the evolution of the rate of feed intake manifests itself progressively. The weight gain, well correlated with the total intake (Table 4), is not correlated with parameters relative to the rate of feed intake. However, a significant negative correlation is observed between acceleration and feed efficiency (−0.57). So, major changes in the feeding pattern based on the rate of feed intake (i.e., higher accelerations) implies a lower feed efficiency. 

In this study, no correlations were found between EST and feed efficiency, which can be explained by the efficiency range and the theory of error propagation. The feeding efficiency recorded in this study shows a small range of variation (0.36 to 0.47, Table 3). The propagation of measurement errors in both the weight of the animals and the intake per visit. Assuming 5% measurement errors in these two variables, the efficiency would accumulate an error of up to 10%, which doubles the coefficient of variation of 5% and thus, cancels the possible differences between animals. Because of that found in this experiment, the results of previous works [14] have not been corroborated in which higher efficiency range (0.082–0.43) correlations of 0.77 between thermal profiles and efficiency were verified.

In an alternative approach, 3-day averages of both thermal parameters and intake parameters have been considered and the correlations between them for each animal have been studied. The intake rate has shown specific relationships with some thermal parameters. Specifically, the difference between the temperature of an animal and the average temperatures of all animals (ΔT) has shown correlations between −0.82 and 0.82 with the intake rate for animals 6205 and 6216, respectively. In the rest of the animals, very different correlation values are observed within these limits, so that the extreme individuals have been studied.

**Figure 13:**
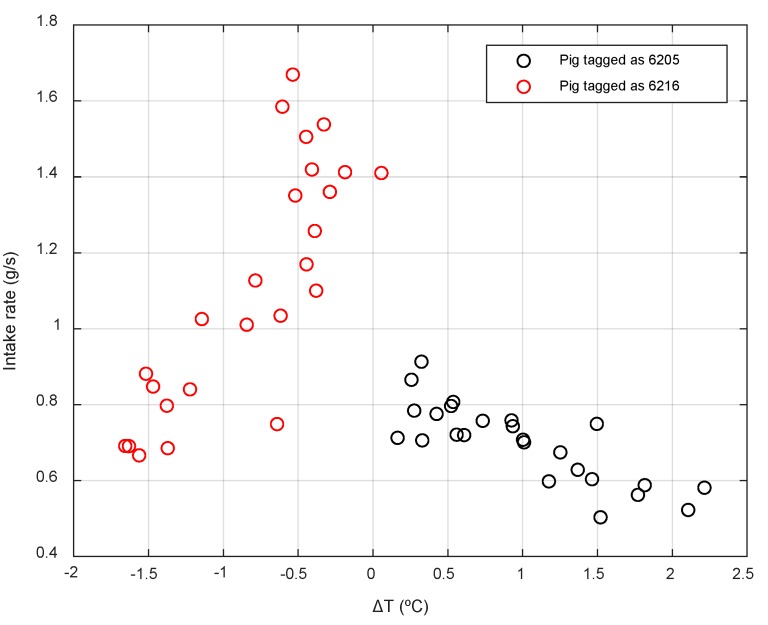
Differences in the individual temperature of animals 6205 and 6216 and the average temperature of the group (ΔT), compared to the intake rate.

Figure 13 shows the plot of ΔT of animals 6205 and 6216 compared to the intake rate (g/s). These animals have opposite behavior patterns. The animal 6205 (r = −0.82) has a temperature always above the average of the group, while the animal 6216 shows a lower temperature than the rest of the group. In addition, the first has a lower intake speed value as well as a lower range (between 0.9 g/s and 0.5 g/s, slow eater), compared to 6216 (between 0.7 g/s and 1.7 g/s, fast eater). The efficiency values were 0.47 and 0.36, respectively (extreme values in the range of efficiencies of this study), showing again that the animals with the greatest changes in feeding patterns based on the rate of feed intake present a lower feed efficiency.

This allows the establishment of connections between the thermal parameters and behavior in the feeding. 

## 4. Conclusions

By registering the high frequency of the temperature of animals in a complete fattening period it is possible to identify individuals with different thermal patterns: animals characterized by a higher temperature and lower thermal variability, compared to animals that register lower average temperatures and greater thermal variability, a pattern that is accentuated during the night. However, the small efficiency range verified in this experiment does not allow the establishment of relations between the thermal profiles and the efficiency itself, as it has been constant in previous studies with wider efficiency ranges.

The parameters extracted from the feeding stations show consistent relationships between the parameters related to the frequency (number of visits), size (total intake, average intake per visit), and duration (total visit time, average visit time) parameters, with absolute values of r greater than 0.83. Based on the frequency of visits and the rate of feed intake, different feeding patterns have been identified among the supervised animals (slow and fast eaters).

The analysis of the number of visits, the feed intake, and the rate of feed intake allowed us to define circadian rhythms in the feeding patterns with more productive intakes during the afternoon.

In spite of the limited range of feed efficiency shown in this study, significant correlations have been verified between the variation of the rate of feed intake along the fattening period and the efficiency (−0.57). It has also been possible to establish a certain connection between the thermal parameters (difference between the temperature of the animal and the mean of the group) with intake parameters (intake rate), showing its potential use in precision phenotyping and animal management, what has to be corroborated in future studies with a greater range of variation in feed efficiency. 

## Figures and Tables

**Table 1:** Animal identification number, initial and final weight of each animal monitored.

Pig No.	Eartag No.	Initial Weight (kg)	Final Weight (kg)
1	6200	41.4	135.0
2	6202	41.8	130.8
3	6204	41.3	134.2
4	6205	43.8	145.6
5	6208	43.0	132.2
6	6209	49.5	165.6
7	6211	43.0	127.9
8	6212	40.0	116.3
9	6213	47.3	138.6
10	6214	47.7	141.9
11	6215	40.6	101.7
12	6216	43.6	119.6
13	6219	35.6	113.3
14	6220	44.0	131.1
15	6221	37.2	111.2
16	6222	37.7	131.2
17	6223	37.0	119.9
18	6224	39.7	119.1
19	6225	37.7	117.1
20	6226	45.0	147.5
21	6227	34.6	113.0
22	6228	44.5	125.7
23	6229	39.0	107.4

**Table 2:** Configuration used in the iButton DS1922E & DS1923 loggers.

Configuration Features	Animal	Environment
Number of loggers	30	6
Capacity (data/iButton)	8192 (8bit)	
Resolution (°C/%HR)	0.5 °C/-	0.5 °C/0.6%
Temperature and Humidity Range	+15 °C to +140 °C-	−20 °C to +85 °C/0 to 100% HR
Sampling interval (s/data)	360	720
Number of iButton replacements	3	

**Table 3:** Values recorded by the feeding station and weighing scale during the test (not considering feeding visits lower than 15 g).

Eartag No.	No. of Visits	Total Visit Time (s)	Average Visit Time (s)	Total Intake (kg)	Average Intake per Visit (kg)	Total Intake Rate (g/s)	Weight Gained (kg)	Efficiency (kg Gained/kg Intake)
**6200**	1036	237,356	229.1	199.9	0.19	0.8423	90.5	0.45
**6202**	632	174,094	275.5	181.7	0.29	1.0436	83.1	0.46
**6204**	1088	233,361	214.5	188.1	0.17	0.8060	83.3	0.44
**6205**	1224	296,799	242.5	207.9	0.17	0.7006	97.1	0.47
**6208**	440	185,895	422.5	182.7	0.42	0.9830	85.8	0.47
**6209**	573	236,342	412.5	245.3	0.43	1.0379	110.6	0.45
**6211**	746	174,392	233.8	169.5	0.23	0.9717	79.7	0.47
**6212**	1082	260,278	240.6	166.4	0.15	0.6393	70.8	0.43
**6213**	1777	286,004	160.9	193.7	0.11	0.6772	86.4	0.45
**6214**	676	262,681	388.6	193.1	0.29	0.7351	91.7	0.47
**6215**	915	204,616	223.6	147.4	0.16	0.7206	56.7	0.38
**6216**	726	180,982	249.3	193.4	0.27	1.0687	70.4	0.36
**6219**	1521	171,262	112.6	175.2	0.12	1.0229	69.1	0.39
**6220**	526	215,338	409.4	204.5	0.39	0.9498	79.5	0.39
**6221**	639	148,928	233.1	161.8	0.25	1.0864	69.4	0.43
**6222**	2375	298,643	125.7	193.5	0.08	0.6481	87.3	0.45
**6223**	548	226,980	414.2	193.1	0.35	0.8508	77.3	0.40
**6224**	555	200,791	361.8	165.7	0.30	0.8252	68.7	0.41
**6225**	1099	290,816	264.6	166.5	0.15	0.5726	72.5	0.44
**6226**	2027	217,002	107.1	218.0	0.11	1.0044	95.7	0.44
**6227**	608	206,297	339.3	184.3	0.30	0.8934	69.8	0.38
**6228**	772	175,488	227.3	176.2	0.23	1.0038	74.2	0.42
**6229**	504	235,903	468.1	154.1	0.31	0.6532	64.6	0.42

**Table 4:** Correlation between parameters obtained of the feeding station.

Parameters of the Feeding Station	NV	TVT	AVT	TI	AIV	TIR	WG	E
Number of visits [NV]	1.00	0.48	−0.81	0.20	−0.84	−0.31	0.25	0.18
Total visit time (s) [TVT]	0.48	1.00	−0.05	0.28	−0.35	−0.83	0.39	0.33
Average visit time (s) [AVT]	−0.81	−0.05	1.00	0.03	0.90	0.00	0.00	−0.04
Total intake (kg) [TI]	0.20	0.28	0.03	1.00	0.21	0.27	0.88	0.25
Average intake per visit (kg) [AIV]	−0.84	−0.35	0.90	0.21	1.00	0.42	0.11	−0.08
Total intake rate (g/s) [TIR]	−0.31	−0.83	0.00	0.27	0.42	1.00	0.12	−0.16
Weight gained (kg) [WG]	0.25	0.39	0.00	0.88	0.11	0.12	1.00	0.67
Efficiency (kg gained/kg intake) [E]	0.18	0.33	−0.04	0.25	−0.08	−0.16	0.67	1.00

**Table 5:** Correlation between the slopes of the feed intake vs. time adjustment lines, acceleration of the rate of feed intake, weight gained, and feed efficiency.

Correlations	Acceleration of Rate Feed Intake	Slope of Feed Intake vs. Time (P1)	Slope of Feed Intake vs. Time (P2)	Slope of Feed Intake vs. Time (P3)	Weight Gained	Efficiency
Acceleration of rate feed intake	1.00	0.29	0.38	0.75	−0.31	−0.57
Slope of feed intake vs. time (P1)	0.29	1.00	0.86	0.72	0.11	−0.14
Slope of feed intake vs. time (P2)	0.38	0.86	1.00	0.81	0.14	−0.08
Slope of feed intake vs. time (P 3)	0.75	0.72	0.81	1.00	−0.07	−0.30
Weight gained	−0.31	0.11	0.14	−0.07	1.00	0.67
Efficiency	−0.57	−0.14	−0.08	−0.30	0.67	1.00
